# PIH1D3-knockout rats exhibit full ciliopathy features and dysfunctional pre-assembly and loading of dynein arms in motile cilia

**DOI:** 10.3389/fcell.2023.1282787

**Published:** 2023-10-12

**Authors:** Tingting Zhang, Shiquan Cui, Xinrui Xiong, Ying Liu, Qilin Cao, Xu-Gang Xia, Hongxia Zhou

**Affiliations:** ^1^ Department of Environmental Health Sciences, Robert Stempel College of Public Health and Social Work, Florida International University, Miami, FL, United States; ^2^ The Center for Translational Sciences, Port St Lucie, FL, United States

**Keywords:** *PIH1 domain-containing protein 3*, cilia, ciliopathy, rat, hydrocephalus, PIH1D3

## Abstract

**Background:** Recessive mutation of the X-linked gene, *PIH1 domain-containing protein 3* (*PIH1D3*), causes familial ciliopathy. PIH1D3 deficiency is associated with the defects of dynein arms in cilia, but how PIH1D3 specifically affects the structure and function of dynein arms is not understood yet. To gain insights into the underlying mechanisms of the disease, it is crucial to create a reliable animal model. In humans, rats, and mice, one copy of the *PIH1D3* gene is located on the X chromosome. Interestingly, mice have an additional, intronless copy of the *Pih1d3* gene on chromosome 1. To develop an accurate disease model, it is best to manipulate the X-linked *PIH1D3* gene, which contains essential regulatory sequences within the introns for precise gene expression. This study aimed to develop a tailored rat model for PIH1D3-associated ciliopathy with the ultimate goal of uncovering the intricate molecular mechanisms responsible for ciliary defects in the disease.

**Methods:** Novel Pih1d3-knockout (KO) rats were created by using TALEN-mediated non-homologous DNA recombination within fertilized rat eggs and, subsequently, underwent a comprehensive characterization through a battery of behavioral and pathological assays. A series of biochemical and histological analyses were conducted to elucidate the identity of protein partners that interact with PIH1D3, thus shedding light on the intricate molecular mechanisms involved in this context.

**Results:** PIH1D3-KO rats reproduced the cardinal features of ciliopathy including situs inversus, defects in spermatocyte survival and mucociliary clearance, and perinatal hydrocephalus. We revealed the novel function of PIH1D3 in cerebrospinal fluid circulation and elucidated the mechanism by which PIH1D3 deficiency caused communicating hydrocephalus. PIH1D3 interacted with the proteins required for the pre-assembly and uploading of outer (ODA) and inner dynein arms (IDA), regulating the integrity of dynein arm structure and function in cilia.

**Conclusion:** PIH1D3-KO rats faithfully reproduced the cardinal features of ciliopathy associated with PIH1D3 deficiency. PIH1D3 interacted with the proteins responsible for the pre-assembly and uploading of dynein arms in cilia, and its deficiency led to dysfunctional cilia and, thus, to ciliopathy by affecting the pre-assembly and uploading of dynein arms. The resultant rat model is a valuable tool for the mechanistic study of PIH1D3-caused diseases.

## Introduction

Cilia, the microtubule-based cellular organelles, are classified as motile cilia, characterized by 9 + 2 axonemes, and primary cilia, characterized by 9 + 0 axonemes. The dynein arms, both inner and outer, are integral constituents of the axonemal structure after they are pre-assembled in the cytoplasm and transported to the axonemes of cilia (Bonnefoy et al., 2018; Lee et al., 2020). The inner dynein arms (IDA) connect adjacent outer doublets, exerting forces pivotal for inducing the bending motion of cilia (Sha et al., 2022; Wang et al., 2023; Wu et al., 2023). Conversely, the outer dynein arms (ODA) are tethered to the A-tubule of each outer doublet and extend outward toward the B-tubule (Ostrowski et al., 2010; Rao et al., 2021). This spatial configuration enables ODA to function as molecular motors, harnessing energy from ATP hydrolysis to generate precise mechanical forces, thereby orchestrating the coordinated and rhythmic beating of cilia (Louvi and Grove, 2011; Zimmermann et al., 2023). The unique structure confers cilia with flexibility and motility. Motile cilia exhibit synchronized beating patterns that facilitate fluid and particle propulsion, playing important roles in cellular processes such as mucociliary clearance in the respiratory tract and cerebrospinal fluid (CSF) circulation in the central nervous system (Louvi and Grove, 2011). In contrast, primary cilia are sensory organelles that do not exhibit motility but serve as crucial sensory antennae, which transduce extracellular signals to intracellular pathways, thereby influencing cell cycle regulation and tissue development (Phua et al., 2017; Anvarian et al., 2019; Kasahara and Inagaki, 2021; Mill et al., 2023). Defects in either motile or primary cilia cause a group of multisystemic human diseases, collectively called ciliopathy.

Ciliopathy can be caused by pathogenic mutations in different genes including *PIH1 domain-containing protein 3* (*PIH1D3*). Recessive mutation in PIH1D3 causes male infertility, situs inversus, and respiratory symptoms. Disease severity depends on the mutation types including deletion, frameshift, and point mutations detected in the *PIH1D3* gene (Olcese et al., 2017; Paff et al., 2017; Aprea et al., 2021), indicating that the preservation of function in mutated PIH1D3 affects phenotypic expression in patients. In humans, rats, and mice, a copy of the multi-exonal *PIH1D3* gene is located on chromosome X (Paff et al., 2017). Interestingly, mice have an additional, intronless copy of the *Pih1d3* gene on chromosome 1 (Dong et al., 2014). Depletion of the intronless *Pih1d3* gene on chromosome 1 causes the only phenotype—male infertility (Dong et al., 2014). In contrast to the intronless *Pih1d3* gene on chromosome 1, the X-linked *PIH1D3* gene is the right target for genetic manipulation to create an accurate disease model because it contains essential regulatory sequences within its introns for precise gene expression.

To assess the functions of PIH1D3 in an intact system, we chose rats rather than mice for modeling PIH1D3 deficiency. Deletion of the X-linked Pih1d3 gene in knockout (KO) rats caused growth retardation and premature death during postnatal ages. PIH1D3-KO rats recapitulated the cardinal features of ciliopathy including hydrocephalus, male infertility, and mucus accumulation in the respiratory tracts. PIH1D3 biochemically interacted with the proteins involved in the pre-assembly and trafficking of outer (ODA) and inner dynein arms (IDA), and PIH1D3 deficiency in KO rats led to ciliary dysfunction and, thus, to ciliopathy by affecting the pre-assembly and uploading of dynein arms.

## Materials and methods

### Generation and breeding of *PIH1D3*-knockout rats

Animal use adhered to the NIH guidelines, and the animal use protocol was approved by the Institutional Animal Care and Use Committee at Florida International University.

Rats were housed at the animal facility of Florida International University. A male paired with a female rat were housed in a ventilated cage with free access to food and water. PIH1D3-KO rats were created using a Sprague–Dawley (SD) strain that was established in the laboratory (Zhou et al., 2009). The rat *Pih1d3* gene consists of eight exons located on the X chromosome. Aligning the human and rat *Pih1d3* gene indicated that exon 5 encodes the conservative domain of PIH1D3 protein, suggesting exon 5 of the rat *Pih1d3* gene to be an appropriate target for gene deletion. The artificial restriction enzyme, transcription activator-like effector nuclease (TALEN), was employed for creating the quick and minimal off-target deletion of the targeted sequence from the *Pih1d3* gene in rats. A pair of effective TALENs (the left: 5′-TCC​TGA​ACC​CAG​GAG​TGA​TG; the right: 5′-TCT​GGA​ACC​TCT​TCT​GGA​TT) were identified targeting exon 5 of the rat *Pih1d3* gene. Synthetic mRNA encoding the Pih1d3 TALENs was injected into the pronuclei of fertilized rat eggs, and the TALEN enzymes translated from the injected mRNA cleaved the targeted DNA to induce non-homologous DNA end-joining repair. Targeted gene modification was detected by PCR with specific primers (forward: 5′-TTC​GTG​ATT​ACA​AGT​AAC​AGT; reverse: 5′-TAA​TAG​TAT​CAG​ATG​ACA​TTC​GTG​A) and was confirmed by subsequent DNA sequencing. A founder female rat was identified carrying the heterozygous deletion of 10 nucleotides (deleted sequence: 5′-AGAACATCTG) from exon 5 of the rat *Pih1d3* gene, and this deletion resulted in a frameshift in the subsequent coding sequence of the rat *Pih1d3* gene, introducing two premature stop codons within exon 6 of the *Pih1d3* gene. The heterozygous Pih1d3-KO female rats were bred with wild-type male rats for more than 20 generations to minimize the potential off-target modification in the rat genome before the KO rats were used for phenotypic expression and characterization.

The day of vaginal plug detection in mating female rats was regarded as the embryonic day 0.5 (E0.5) of embryo age. The day of the birth of newborn rats was considered postnatal day zero (P0), relating to newborn rat age. Newborn rats were counted at 9:00 every morning to calculate survival rates. Tissues of dead rats were collected for analyzing genotypes. Newborn rats were weighed in the morning from P7 onward. Surviving newborn rats were ear-tagged and tail-clipped on P14 for genotyping by PCR. PIH1D3-KO rats and WT male littermates were paired for biochemical and histological analyses.

### Cerebrospinal fluid flow analysis

An enlarged head was the evident phenotype of Pih1d3-KO rats at birth. KO rats and WT male littermates at age P3 were anesthetized by inhaling isoflurane and placed on a stereotaxic frame. The left lateral ventricle (LV) of the rats was visible under light, and Evans blue diluted in PBS to 2% working concentration was injected into the left ventricle. A measure of 2 µL of Evans blue was injected through a glass syringe into each LV. The glass syringe was left in place for 1 min after injection to prevent the leakage of dyes through injection holes. The rats were placed onto a warming pad after injection and were constantly inhaling isoflurane. The rats were euthanized 15 min after Evans blue injection. Rat brains were fixed in 4% paraformaldehyde at 4°C overnight and then cut into coronal sections for photographing under a stereo microscope (Leica DFC295).

### Antibody information

The following primary antibodies were used: rabbit polyclonal anti-PIH1D3 (Proteintech, 25309-1-AP); mouse monoclonal anti-acetylated alpha-tubulin IgG1 (Proteintech, CL488-66200); rabbit polyclonal anti-DNAI1 (Abcam, ab171964); rabbit polyclonal anti-DNAI2 (Proteintech, 17533-1-AP); rabbit polyclonal anti-DNALI1 for immunofluorescence staining (Proteintech, 17601-1-AP); mouse monoclonal anti-DNALI1 for immunoblotting (Santa Cruz, Sc-514831); rabbit polyclonal anti-IFT52 (Proteintech, 17534-1-AP); rabbit polyclonal anti-IFT57 (Proteintech, 11083-1-AP); rabbit polyclonal anti-PIH1D1 (Proteintech, 19427-1-AP); rabbit polyclonal anti-IFT20 (Proteintech, 13615-1-AP); rabbit polyclonal anti-IFT81 (Proteintech, 11744-1-AP); mouse monoclonal anti-Flag (Novus, NBP1-97410); and mouse monoclonal anti-Myc (Invitrogen, MA1-21316). Primary antibodies were diluted at 1:1,000 or 1:500 for immunoblotting and used for immunostaining at the lowest dilutions recommended by the manufacturers. Anti-mouse secondary antibody (Molecular Probes, Alexa Fluor 488) and anti-rabbit secondary antibody (Molecular Probes, Alexa Fluor 594) were diluted at 1:1,000 for immunofluorescence staining.

### Histology and immunostaining

The rats were perfused with 4% paraformaldehyde after they were deeply anesthetized with a ketamine (10 mg/mL)/xylazine (1 mg/mL) mixture as previously described (Yuan et al., 2021). Rat tissues and timed embryos were harvested and further fixed in 4% paraformaldehyde overnight at 4°C. Tissues were dehydrated in a series of ethanol solutions (70%, 80%, 95%, and 100%), cleared twice in xylene, and immersed and embedded in paraffin. Paraffin-embedded tissue blocks were sectioned at 5 µm thickness on the Leica RM 2135 microtome. Tissue sections were dewaxed in xylene and rehydrated in a series of ethanol solutions. Rehydrated tissue sections were stained with hematoxylin and eosin (H&E staining) and were observed and photographed under a Nikon light microscope.

For immunohistochemistry and immunofluorescence staining, rehydrated tissue sections were first incubated with the blocking solution at room temperature for 30 min and then incubated with primary antibodies diluted to the desired concentration at 4°C overnight. After washing off unbound primary antibodies, the tissue sections were incubated with the fluorescence or enzyme-conjugated secondary antibodies at room temperature for 1 h. Unbound secondary antibodies were washed off with PBS, and tissue sections were mounted using a ProLong Antifade Mountant with DAPI (Invitrogen, 2188179). Tissue sections were assessed for gene expression by immunohistochemistry using a Nikon microscope or by immunofluorescence staining using a confocal microscope (Nikon confocal system). Single-layer images were scanned, and then image Z-stacks (at 1 mm intervals) were projected to reconstruct cell structures.

### TUNEL assay

Apoptotic cell death in rat testes was assessed using an *in situ* cell death detection kit (Roche, Germany). Paraffin-embedded tissue sections were first dewaxed and rehydrated and then incubated with a TUNEL reaction mixture at 37°C for 1 h. TUNEL-positive cells were observed under a fluorescence microscope, and six randomly selected fields were counted for all TUNEL-positive cells. The ratio of TUNEL-positive cells to the total cells in the selected fields was calculated as a percentage of apoptotic cells.

### Transmission electron microscopy

Rats were transcardially perfused with 4% paraformaldehyde under deep anesthesia with ketamine (10 mg/mL)/xylazine (1 mg/mL). The rat trachea was dissected using a sharp surgical knife, and the tissues were post-fixed in the same fixative overnight at 4°C. Fixed tissues were embedded in Epon 812 and were cut into ultrathin sections using a diamond knife. The ultrathin tissue sections were transferred onto copper grids (50 mesh) coated with a Formvar membrane and stained with uranyl acetate and lead citrate. The stained sections were observed and photographed using an electron microscope (Hitachi HT7700).

### Cell culture and transfection

HEK293 cells purchased from ATCC (CRL-11268TM) were cultured in DMEM supplemented with 10% fetal bovine serum (FBS). Cells were transfected with plasmids premixed with Lipofectamine 2000 as previously described (Wu et al., 2015). Cells were transfected in the absence of FBS for 4 h and supplemented with 10% FBS thereafter. Cells transiently transfected with a desired combination of plasmids were harvested 48 h after transfection. Control cells were sham-transfected with a carrier plasmid expressing no mammalian cell gene. An individual plasmid expressing a Myc-tagged target protein was co-transfected with a plasmid expressing a PIH1D3 peptide into HEK293 cells. Each Myc-tagged protein was individually assessed for its interaction with the PIH1D3 peptide. HEK293 cells were transiently transfected with plasmids and not modified for stable transgene expression. No authentication was performed to characterize the transfected cells.

### Immunoblotting and immunoprecipitation

For immunoprecipitation, cells were initially lysed in lysis buffer (Promega) and were fully broken by sonication as described previously (Xia et al., 2013). The lysates were cleared by centrifugation for 10 min at 4°C, and 500 µg of total protein per sample was incubated with FLAG (Sigma) or MYC (Thermo Fisher)-binding resin to precipitate tagged proteins and affiliated protein partners. Bound proteins were eluted with SDS sample buffer for MYC beads and with FLAG peptides for FLAG beads. Eluted proteins were boiled for 10 min to dissociate protein complexes and were detected by immunoblotting with specified antibodies.

For immunoblotting, animal tissue and HEK293 cells were homogenized in RIPA buffer, and proteins in the lysates were resolved by SDS-PAGE (Zhou et al., 2010). Immunoreactivity for a protein was detected with specified primary antibodies after the resolved proteins were transferred onto nitrocellulose membranes.

### Statistical analysis

Apoptotic cells in rat testes were compared between groups of rats with different genotypes. Unpaired *t*-tests were used to analyze any differences in counts between groups using GraphPad InStat software. A *p*-value <0.05 was considered statistically significant. The data were not assessed for normality, and no test for outliers was conducted.

## Results

### Deletion of Pih1d3 in rats caused growth retardation and postnatal lethality

**FIGURE 1:**
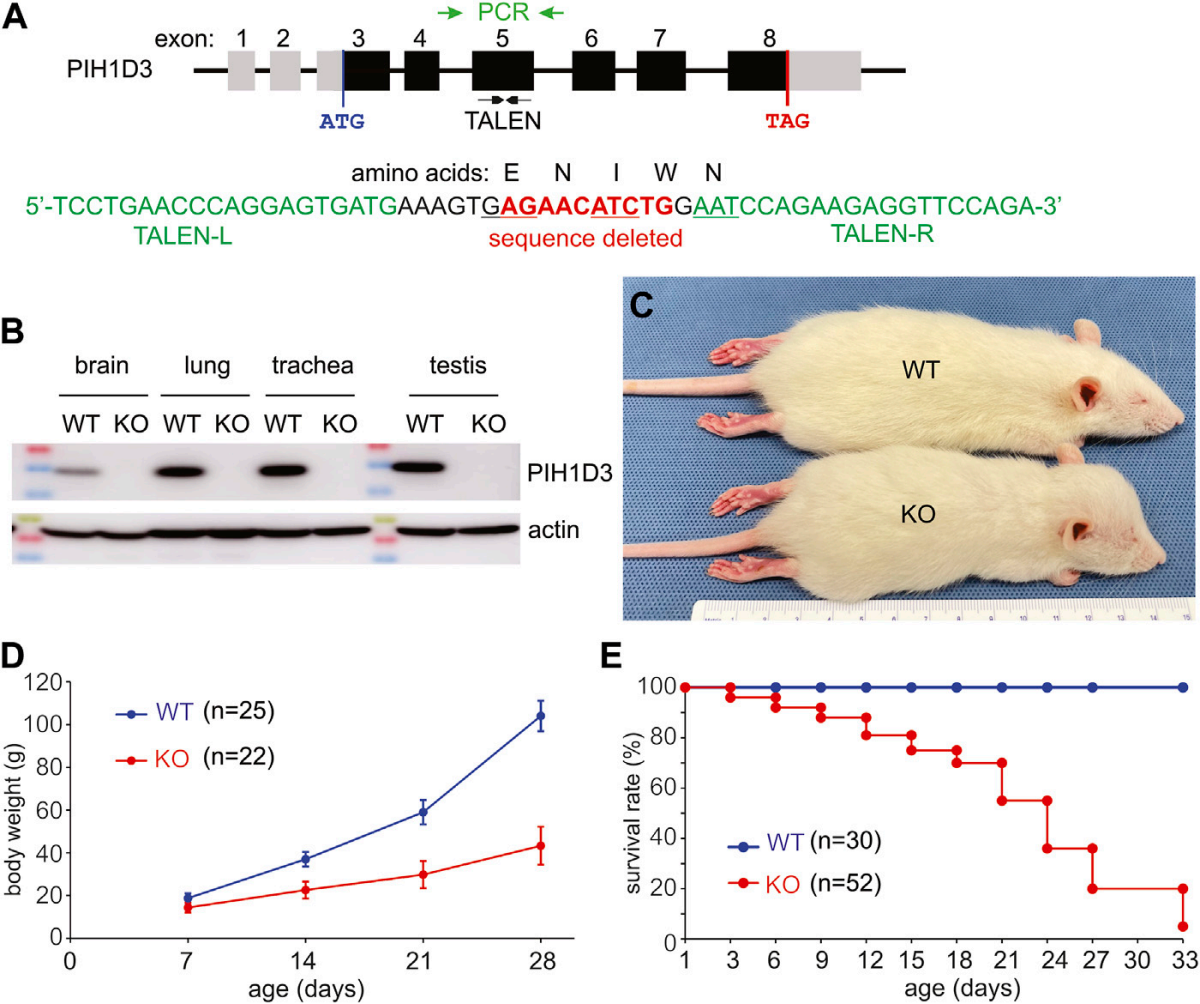
Deletion of the *Pih1d3* gene in knockout rats causes growth retardation and premature death. **(A)** Schematic showing the structure of the rat *Pih1d3* gene with the region targeted for mutagenesis by TALEN and with the region amplified by PCR for genotyping and sequencing (gene structure not drawn to scale). Partial sequence of exon 5 on the rat *Pih1d3* gene shows the binding sites for the left (TALEN-L) and right (TALEN-R) arms of an effective TALEN as well as the sequence deleted in the Pih1d3-KO rats. Deletion of 10 nucleotides from exon 5 results in the shifting of the open reading frame, introducing two consecutive stop codons within exon 6 of the rat *Pih1d3* gene (sequence verified, but data not shown). **(B)** Immunoblotting revealed that the PIH1D3 protein was depleted from the tissues of Pih1d3-KO rats. Equal loading of total proteins (20 µg/lane) was assessed by immunoblotting on the same membrane for actin. Tissues were dissected from Pih1d3-KO male rats and their WT male littermates at the age of 15 days. **(C)** A representative image shows that a Pih1d3-KO rat had a smaller body but an enlarged head compared to its male WT littermate at the age of 28 days. **(D)** Growth chart showing the retarded growth of body weight (g, gram) in Pih1d3-KO rats compared to their male WT littermates (data were the means ± standard deviation). **(E)** Kaplan–Meier survival analysis showing the premature death of Pih1d3-KO rats during the postnatal development stage. Very few KO rats survived beyond 30 days, but none of them survived by 70 days of ages.

Pih1d3 is an X-linked gene consisting of eight exons and spanning 50 K nucleotides in the rat genome (Figure 1). PIH1D3 mRNA and protein were expressed robustly in the reproductive organs, including the ovary and testis, substantially in the respiratory tracts, including the nasal cavity, trachea, and lung, and in the brain of postnatal rats (Figure 1; Supplementary Figure S1). Injection of TALEN-encoding mRNA into fertilized eggs successfully deleted a fragment of 10 nucleotides from exon 5 of the *Pih1d3* gene (Figure 1A), resulting in a frameshift within the coding sequence of the *Pih1d3* gene. After conducting a breeding program spanning more than 20 generations, we consistently observed the stable transmission of the Pih1d3-KO allele across successive generations. This transmission was confirmed through the absence of the PIH1D3 protein, as detected by immunoblotting (Figure 1B). Notably, Pih1d3 hemizygous KO male rats (referred to as KO males) exhibited significant slow growth during postnatal development compared to wild-type (WT) littermates (Figures 1C, D). Furthermore, all Pih1d3 hemizygous male rats did not survive past the postnatal development stage, failing to reach sexual maturity. Therefore, we were unable to obtain homozygous female rats carrying the Pih1d3-KO allele (referred to as KO females), which limited our analyses exclusively to hemizygous male rats.

### Pih1d3 deficiency caused communicating hydrocephalus in KO rats

**FIGURE 2:**
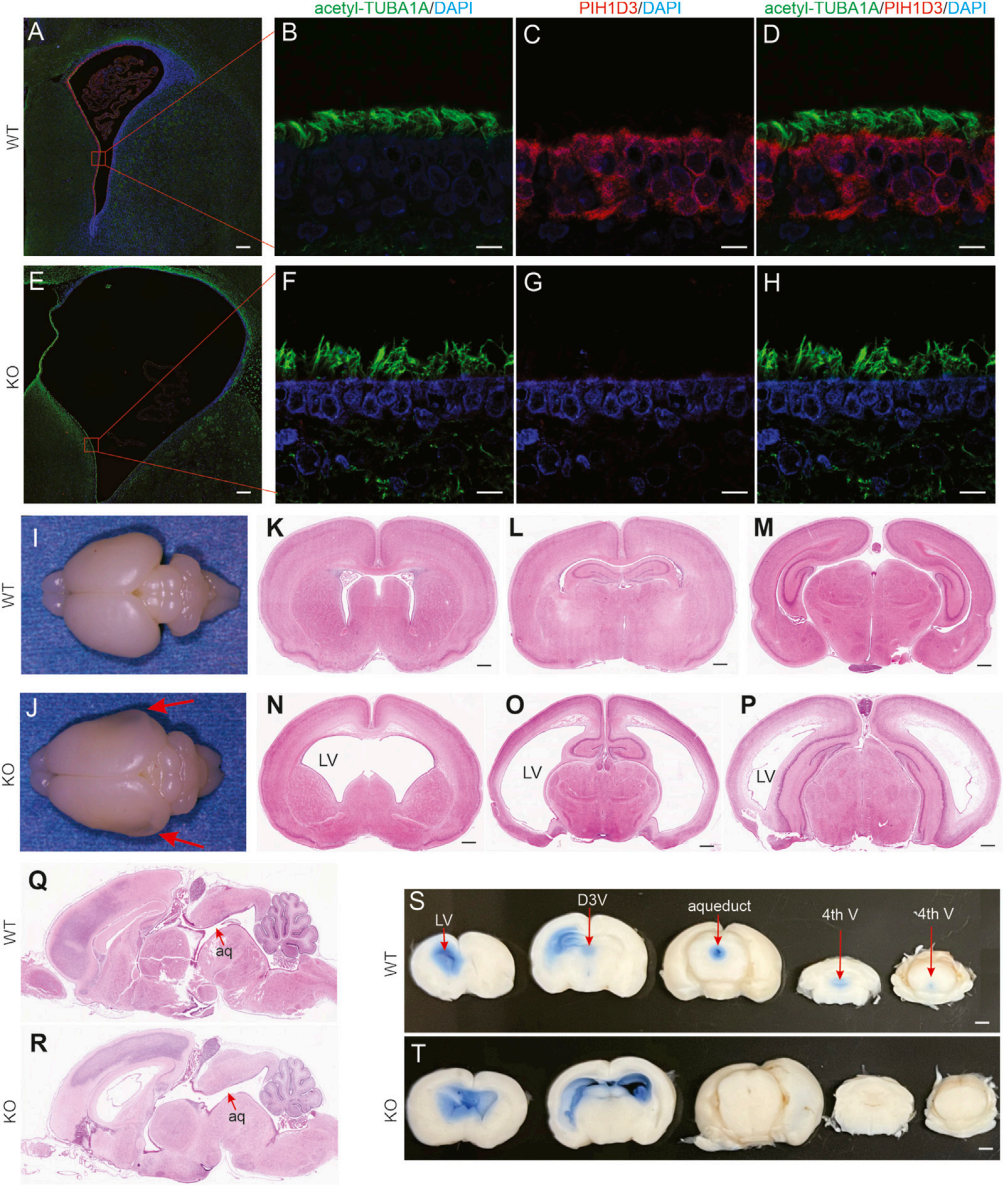
Communicating hydrocephalus detected in Pih1d3-KO rats. **(A–H)** Immunofluorescent staining for acetylated alpha-tubulin (acetyl-TUBA1A, a marker for motile cilia) and PIH1D3 reveals the depletion of PIH1D3 proteins from ependymal cells in Pih1d3-KO rats. KO rats and WT male littermates were examined at the age of 5 days. Scale bars: 100 µm **(A,E)** and 30 µm **(B–D,F–H)**. **(I,J)** Images of Pih1d3-KO and WT rat brains at postnatal day 5. Arrows point to the enlarged and transparent brain hemispheres. **(K–P)** HE staining of coronal brain sections reveals enlarged lateral ventricles (LV). Scale bar: 150 µm. **(Q,R)** HE staining of sagittal brain sections reveals no blocking of the aqueduct (aq). **(I-J)** The same rat brains were sectioned for HE staining **(K–R)**. **(S,T)** CSF flow analysis reveals that Evans blue injected into the left LV flowed into the right LV but not into the dorsal third ventricle (D3V), aqueduct, and the fourth ventricle (fourth V) in Pih1d3-KO rats. By contrast, Evans blue flowed from the left LV into D3V, aqueduct, and then fourth V but not into the right LV in wild-type rats. Scale bar: 200 µm.

In the brain, PIH1D3 was mainly expressed in ependymal cells and choroid plexus epithelial cells (Figure 2; Supplementary Figure S2), the cells that are involved in the production and mobilization of cerebrospinal fluid (CSF). Pih1d3 deficiency may impair CSF production and circulation. Indeed, Pih1d3-KO rats exhibited enlarged heads as one prominent phenotype (Figure 1C). Hydrocephalus was detected in Pih1d3-KO rats since embryonic stages and was more severe during postnatal stages (Figure 2; Supplementary Figure S2). Anatomical assessment revealed no blocking of CSF circulation paths in the brains (Figure 2). CSF circulation assay revealed that Evans blue injected into one lateral ventricle flowed down into the third ventricle and aqueduct in WT rats (Supplementary Figure S2). By contrast, the dye did not flow into the third ventricle and aqueduct but instead into the contralateral ventricle in Pih1d3-KO rats (Figure 2T). Evans blue assay suggested that CSF mobilization was impaired in Pih1d3-KO rats. Pih1d3 deficiency caused communicating hydrocephalus in Pih1d3-KO rats. Hydrocephalus was consistently observed in all KO rats examined.

### Pih1d3 deficiency in KO rats caused situs inversus and accumulation of mucus in respiratory tracts

In Pih1d3-KO rats, organs in the chest and abdomen cavities were positioned on the opposite sides compared to those in WT littermates (Supplementary Figure S3). We examined 60 newborn KO rats, 27 of which presented situs inversus. The frequency of situs inversus in Pih1d3-KO rats was 45%, close to the frequency reported in patients.

**FIGURE 3:**
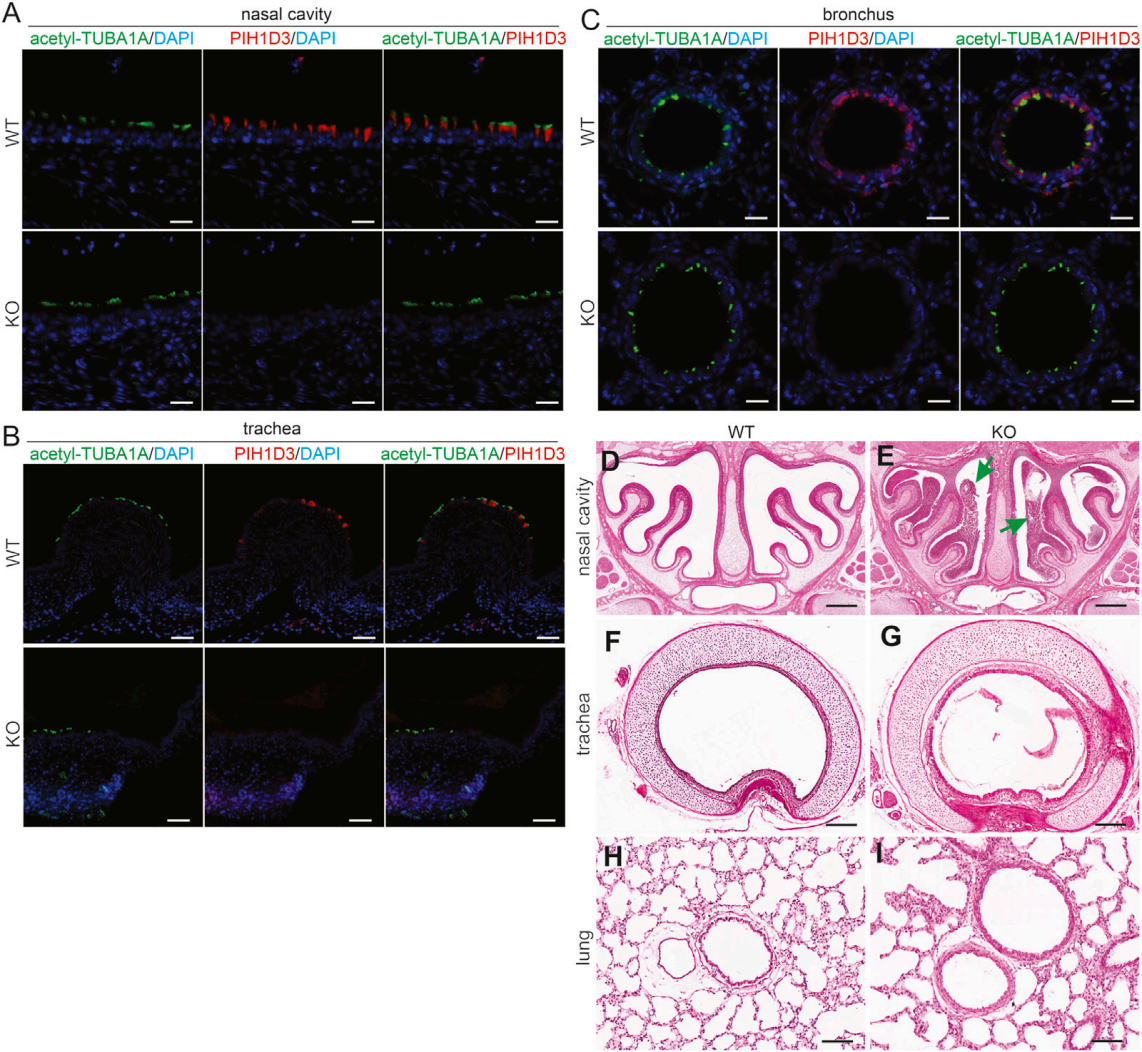
Accumulation of mucus in the upper respiratory tract revealed in Pih1d3-knockout rats. **(A–C)** Immunofluorescent staining for acetylated alpha-tubulin (acetyl-TUBA1A) and PIH1D3 reveals the depletion of PIH1D3 proteins from the epithelial cells of respiratory tracts in Pih1d3-KO rats compared to WT littermates. Scale bar: 40 µm **(A–C)**. **(D–I)** HE staining reveals that mucus accumulated severely in the nasal cavity (arrows pointed to) and barely in the trachea and bronchus of Pih1d3-KO rats, but not in the WT rats. KO rats and male littermates were examined at the age of 5 days. Scale bar: 200 µm.

In the respiratory system, Pih1d3 was primarily expressed in the ciliated epithelial cells of the nasal cavity, trachea, and bronchus (Figures 3A–C), consistent with the finding in the ependymal cells and choroid plexus epithelial cells of the brain (Figures 2A–H; Supplementary Figure S2). Deletion of Pih1d3 in KO rats caused the accumulation of mucus in the upper respiratory tract, particularly in the nasal cavity (Figures 3D–I). In contrast to the incomplete penetrance of situs inversus, the phenotypes of the respiratory system were consistently observed in all KO rats examined.

### Deletion of Pih1d3 in KO rats impaired spermatogenesis

**FIGURE 4:**
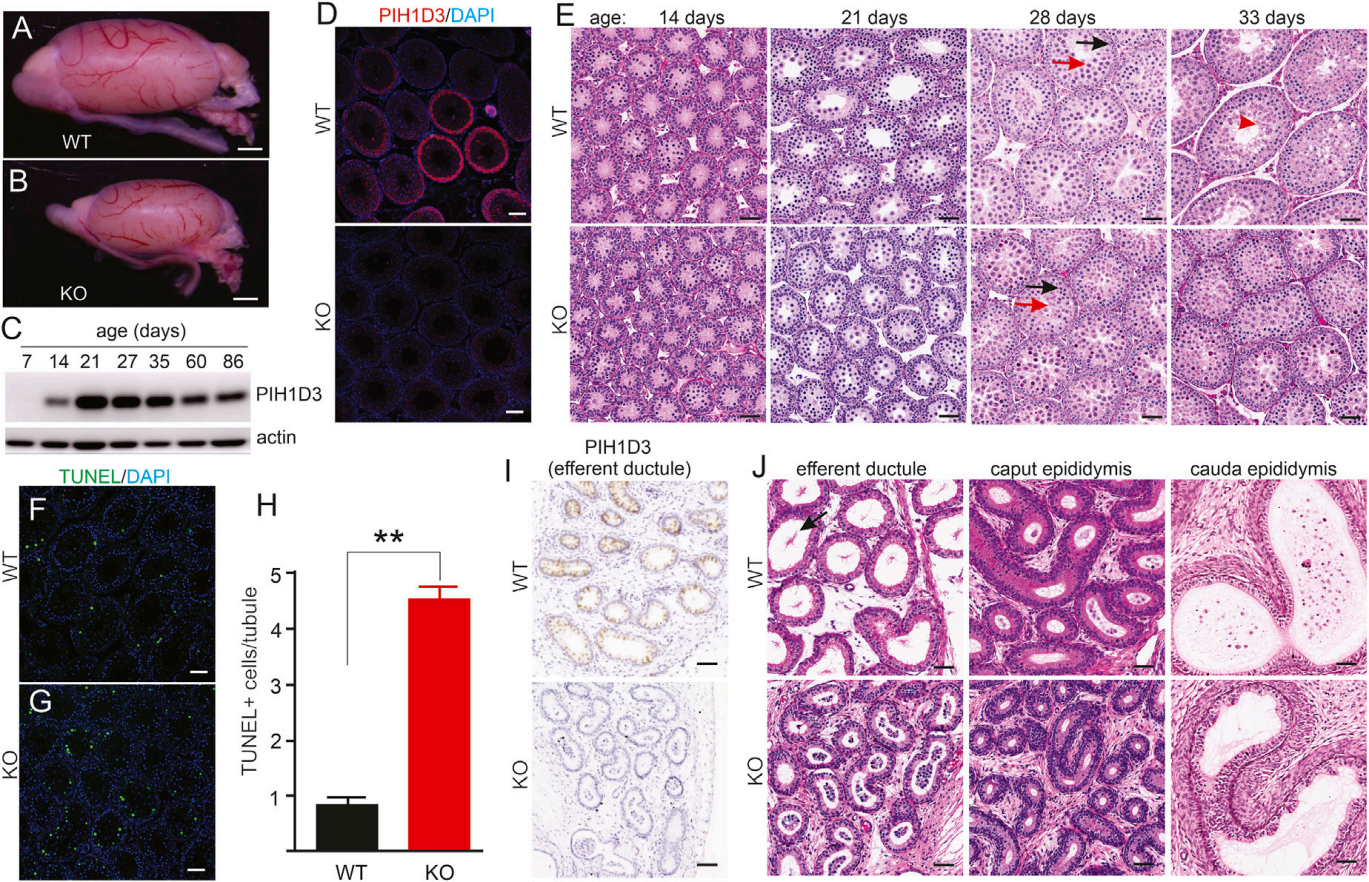
Spermatogenesis impaired in Pih1d3-KO rats. **(A,B)** Images of the testis taken from Pih1d3-KO and WT rats at the age of 28 days. Scale bar: 500 µm. **(C)** Immunoblotting reveals the expression of PIH1D3 in the rat testis from postnatal day 7 onward. **(D)** Immunofluorescent staining reveals PIH1D3 depleted from the spermatogenic cells of the KO rat testis. Scale bar: 40 µm. **(E)** HE staining of seminiferous tubules from KO and WT littermates at varying postnatal (P) ages. At postnatal day 14 (P14), germ cells in both the WT and KO rats were developed into pachytene spermatocytes, and no difference was observed between KO and WT rats. By P27, seminiferous tubules were filled with round spermatids (red arrow) and pachytene spermatocytes (dark arrow) in WT rats, but much fewer round spermatids were observed in KO rats. By P33, elongated spermatids (red arrowhead) were observed in the tubules of WT rats, but not of KO rats. The total number of cells, including pachytene spermatocytes and round spermatids, was significantly decreased in KO rats compared to WT littermates by P33. Scale bar: 50 µm. **(F–H)** TUNEL staining revealed that apoptotic cell death was increased in the seminiferous tubules of KO rats aged P28 compared to WT littermates. Data were the means ± SD (n = 50). **p < 0.01. **(I)** Immunohistochemistry reveals that PIH1D3 was depleted from the epithelium of efferent ductules in KO rats. **(J)** HE staining reveals the occlusion and agglutination of efferent ductules and the abnormal ducts with a narrow lumen in KO rats compared to WT rats at age P35. Few spermatozoa with long tails (arrow) were observed in WT but not in KO rats. Scale bar: 40 µm **(F,G,I,J)**.

Pih1d3-KO rats exhibited a smaller testis than WT littermates during postnatal development (Figures 4A, B). The PIH1D3 protein was detected from postnatal day 14, and its expression reached a plateau by postnatal day 21 (Figure 4C). Immunofluorescent staining revealed that PIH1D3 was mainly expressed in the spermatogenic cells of the testis (Figure 4D). Depletion of Pih1d3 in KO rats impaired spermatid development. The pachytene spermatocytes and round spermatids were significantly decreased in the testis of Pih1d3-KO rats (Figure 4D). TUNEL assay revealed that apoptotic cell death in the seminiferous tubules was significantly increased in Pih1d3-KO rats (Figures 4F–H). Pih1d3 was expressed in the ciliated cells of efferent ductules (Figure 4I). Compared to WT rats, KO rats exhibited the occlusion of efferent ductules and abnormal ducts with a narrow lumen. Pih1d3 deficiency in KO rats impaired spermatogenesis, partly by inducing apoptotic cell death. Cell death is likely ciliary-independent because PIH1D3 contains a PIH domain that is associated with apoptotic cell death (Inoue et al., 2010).

### Deletion of Pih1d3 in KO rats disrupted the assembly of inner and outer dynein arms in cilia

**FIGURE 5:**
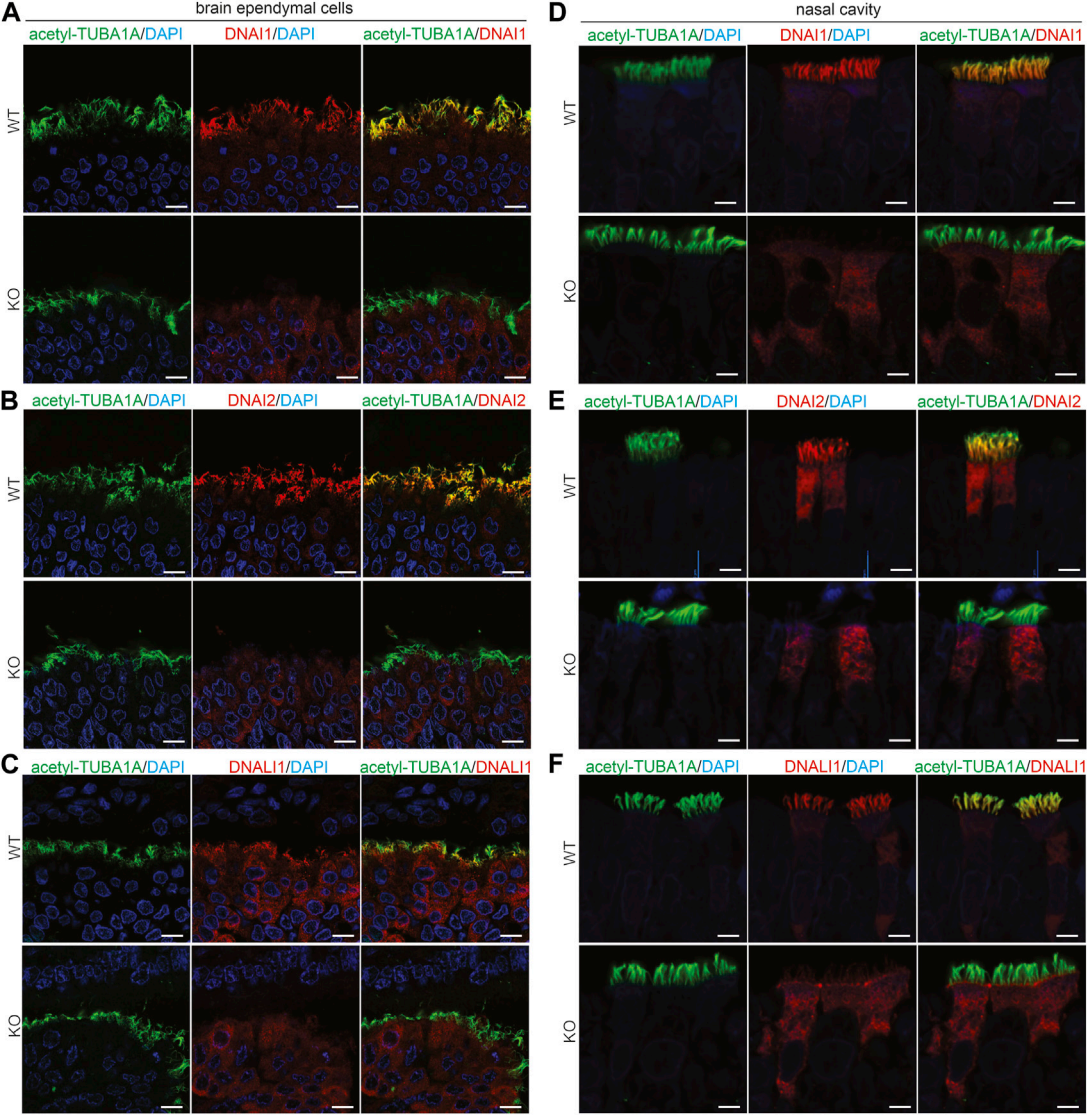
PIH1D3 deficiency results in the depletion of DNAI1, DNAI2, and DNALI1 from motile cilia. **(A–F)** Immunofluorescent staining assessed the colocalization of acetylated alpha-tubulin (acetyl-TUBA1A, a marker for motile cilia) with DNALI1 (marker for inner dynein arms) and with DNAI1 and DNAI2 (markers for outer dynein arms) in the brain ependymal cells and nasal epithelial cells of Pih1d3-KO rats and WT littermates. Acetyl-TUBA1A was well colocalized with DNALI1, DNAI1, and DNAI2 in WT rats but not in the KO rats. Brain tissues were taken from rat embryos at E19, and nasal tissues were taken from postnatal rats at P5. Scale bar: 30 µm.

**FIGURE 6:**
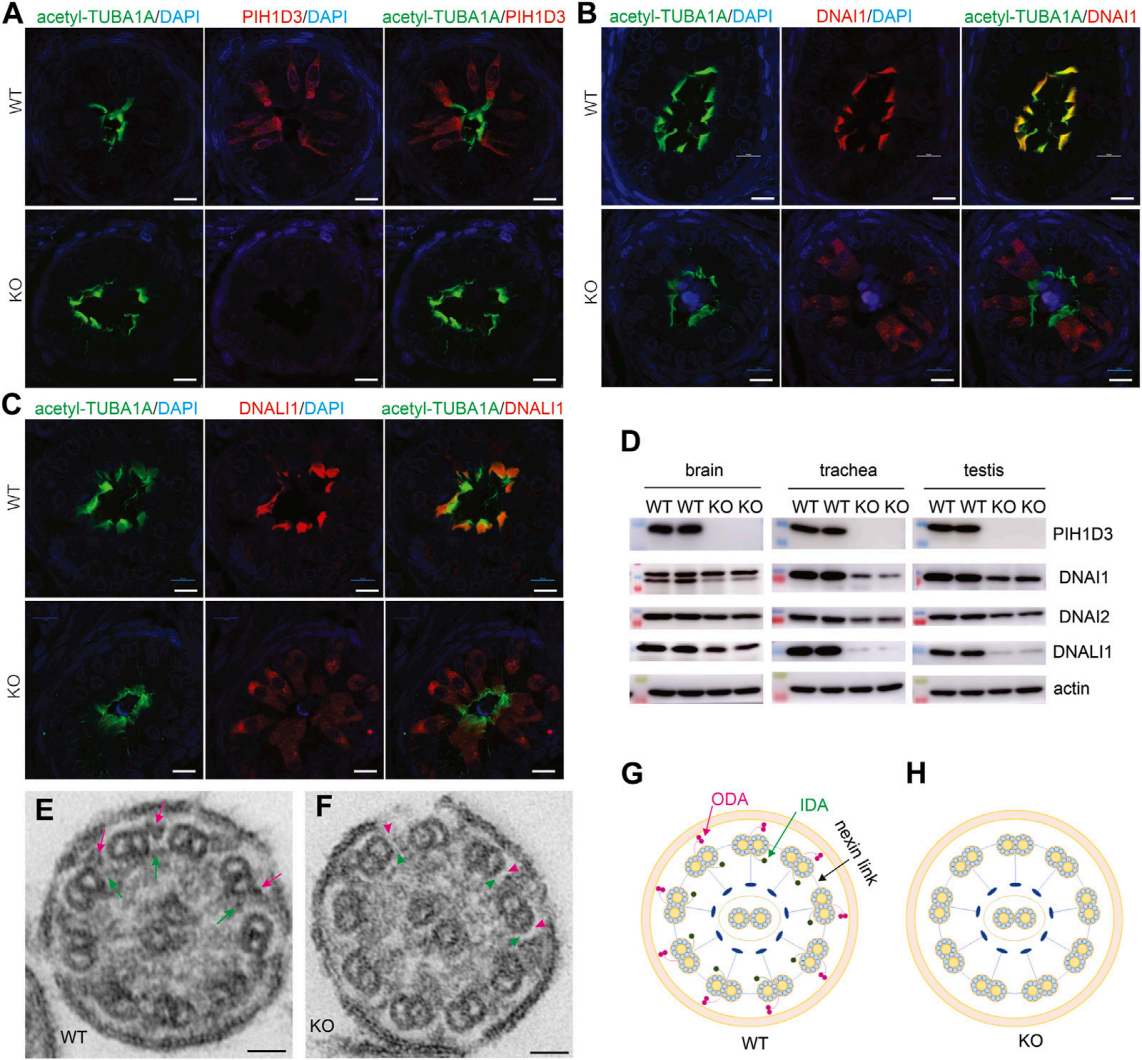
Defective ODA and IDA in the motile cilia of PIH1D3-KO rats. **(A–C)** Immunofluorescent staining reveals that PIH1D3 deficiency in KO rats results in the dislocation of DNAI1 and DNALI1 from motile cilia labeled with acetyl-TUBA1A. The efferent ductules were dissected from Pih1d3-KO rats and WT littermates at the age of 27 days. Scale bar: 30 µm. **(D)** Immunoblotting shows that the expression of the ODA markers DNAI1 and DNAI2 and the IDA marker DNALI1 was reduced in the tissues of Pih1d3-KO rats compared to WT littermates. Rats of age postnatal day 20 were used for dissecting the trachea and testis, and rats of age postnatal day 0 were used for dissecting brains. Equal loading of 20 µg total protein per lane was verified by immunoblotting for actin. Quantification for the density of immunoblotting is shown in Supplementary Figure S4. **(E,F)** Transmission electron microscopy reveals that ODA and IDA were defective in Pih1d3-KO rats. Red arrows point to ODA and green arrows point to IDA in WT rats, whereas red arrowheads point to the absence of ODA and green arrowheads point to the absence of IDA in Pih1d3-KO rats. Tracheas dissected from KO and WT rats aged 20 days were examined by transmission electron microscopy. Scale bar: 20 nm. **(G,H)** Diagrams showing the structure of motile cilia. Compared to WT rats, ODA and IDA were defected in Pih1d3-KO rats.

Pih1d3 was primarily expressed in the epithelial cells distributed across cerebral ventricles, respiratory tracts, and testicles, and its deficiency indeed affected the cellular functions of epithelial cells in KO rats (Figures 1–4). Deletion of Pih1d3 in KO rats disrupted the assembly of dynein arms in the cilia of epithelial cells. Dynein arms consist of inner and outer parts which are identifiable by immunoreactivity for the inner dynein marker DNALI1 and for the outer dynein arm markers DNAI1 and DNAI2, respectively. Deletion of Pih1d3 in KO rats led to the depletion of DNAI1, DNAI2, and DNALI1 from the cilia of epithelial cells in the cerebral ventricles, respiratory tracts, and efferent ductules (Figures 5, 6). Consistently, immunoblotting revealed that the expression of these dynein arm proteins was decreased in the tissues (Figure 6D; Supplementary Figure S4). Transmission electron microscopy confirmed that both the outer and inner dynein arms were defected in KO rats (Figure 6F; Supplementary Figure S5). By contrast, the immunoreactivity for anterograde trafficking-associated proteins, including IFT20, IFT52, and IFT81, was enhanced in the cilia of epithelial cells (Supplementary Figure S6). While IFT52 and IFT81 are the components of the IFT-B1 subcomplex, IFT20 and IFT57 are the components of the IFT-B2 subcomplex. Using the acetyl-TUBA1A antibody for cilia staining allowed for the discrimination of target protein expression patterns. Notably, our observations revealed a pronounced and concentrated staining of the target proteins, specifically IFT20, IFT52, and IFT81, predominantly localized at the distal tips of cilia (Supplementary Figures S6–S8). Some intraflagellar transport proteins examined, including IFT52 and IFT81, were upregulated in the specified tissues of Pih1d3-KO rats (Supplementary Figure S9). The data suggest that a compensatory mechanism was initiated in anterograde trafficking within cilia impacted by Pih1d3 deficiency and that both the enhanced expression and the redistribution of target proteins contribute to the compensatory effects.

### PIH1D3 biochemically interacted with the proteins involved in dynein arm assembly and trafficking

**FIGURE 7:**
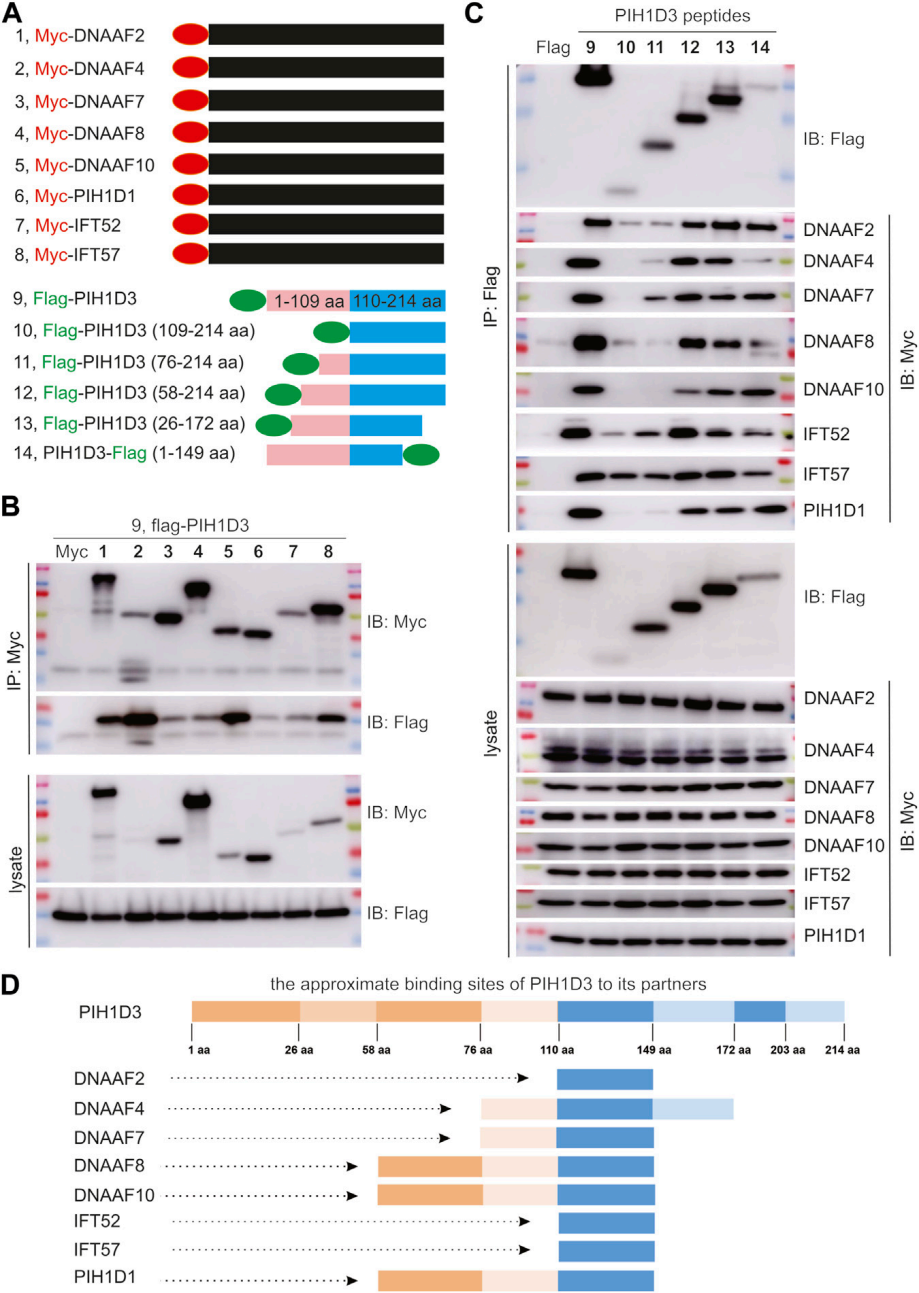
PIH1D3 protein partners revealed by immunoprecipitation (IP) combined with immunoblotting (IB). **(A)** Schematics showing the construction of plasmids expressing Myc-tagged partner proteins and flag-tagged full-length and fragmented PIH1D3 peptides. **(B)** Plasmid expressing that an individual partner protein was co-transfected with a PIH1D3-expressing plasmid into HEK293 cells, and the cell lysates were analyzed by IP and IB 48 h after transfection. **(C)** Plasmids expressing full-length or fragmented PIH1D3 peptides were co-transfected individually with a partner-expressing plasmid into HEK293 cells. The binding affinity of each PIH1D3 peptide for partner proteins was assessed by IP and IB. **(D)** Schematics showing the approximate binding sites on PIH1D3 for each partner protein examined.

The dynein arms of cilia were negatively impacted by Pih1d3 deficiency and the anterograde trafficking proteins were accumulated in the tips of cilia as a compensatory mechanism (Figure 6; Supplementary Figures S6–S9), indicating that PIH1D3 is involved in the assembly of dynein arms. The impact of PIH1D3 on dynein arm assembly at the molecular level is not clear. Immunoprecipitation combined with immunoblotting revealed that PIH1D3 interacted with DNAAF family members, including DNAAF2, DNAAF4, DNAAF7, DNAAF8, and DNAAF10, and with IFT-B complex proteins, including IFT52 and IFT57 (Figure 7). The binding sites on PIH1D3 for individual protein partners were defined accordingly (Figures 7C, D). DNAAF proteins regulate dynein arm assembly, and IFT-B proteins are involved in the anterograde trafficking of dynein arms to cilia. PIH1D3 biochemically interacted with these proteins to impact the assembly and trafficking of dynein arms in ciliated cells. PIH1D1 and PIH1D3 both possess PH domains. The PH domain of PIH1D1 is known to play a role in apoptotic cell death (Inoue et al., 2010). Our analysis revealed that PIH1D3 and PIH1D1 interacted biochemically and are part of a functionally related protein complex. These results suggest that PIH1D1 and PIH1D3 likely share similar functions, possibly related to apoptosis.

## Discussion

We reported a novel rat model that was depleted of PIH1D3 expression and developed the cardinal features of PIH1D3-related diseases (Olcese et al., 2017; Paff et al., 2017; Paff et al., 2018; Aprea et al., 2021; Lennon et al., 2022). PIH1D3-KO rats exhibited situs inversus, defects in spermatocyte survival and mucociliary clearance, and perinatal hydrocephalus. These phenotypes were correlated with the expression of PIH1D3 in affected organs. PIH1D3 was predominantly expressed in the ciliated epithelial cells covering respiratory tracts, testis tubules, ependyma, and choroid plexus. The correlation of the affected organs with PIH1D3 expression suggested that the disease phenotypes in PIH1D3-KO rats resulted from PIH1D3 deficiency. PIH1D3-KO rats were bred with WT rats for more than 10 generations before disease phenotypes were assessed. The off-target effect of TALEN-mediated gene modification, if any, was minimized. During the breeding process, the phenotypes reported, except situs inversus, were consistently observed in all PIH1D3-KO rats examined, indicating that the disease phenotypes caused by PIH1D3 deficiency were reproducible in the KO rats.

**FIGURE 8:**
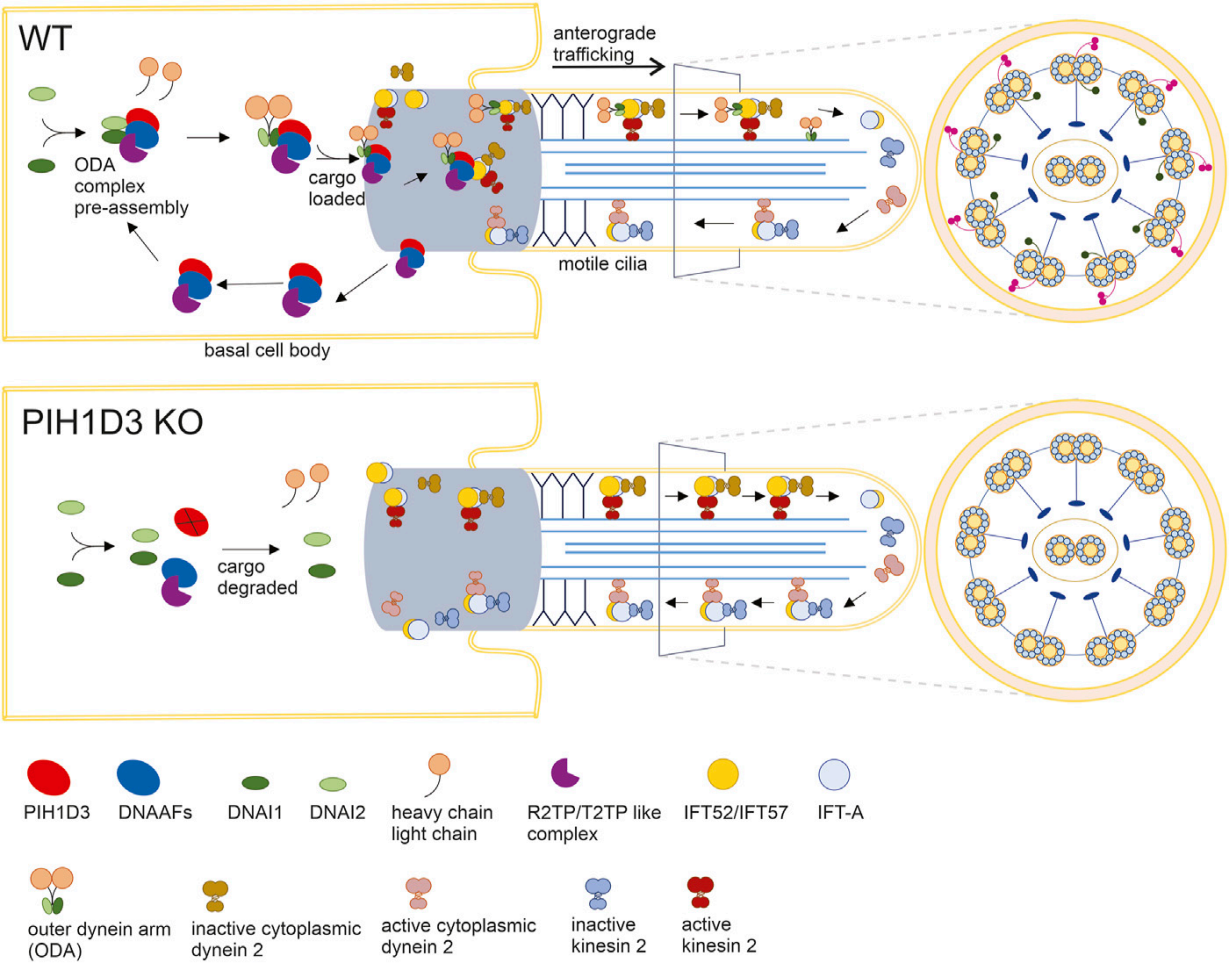
Defected assembling and loading of ODA in KO rats. In WT rats, 1) PIH1D3 functions as a critical part of the protein complex mediating the pre-assembly and assembly of ODA through interacting with the DNAAF family and 2) PIH1D3 participates in the loading of the ODA complex onto vehicles for anterograde trafficking to ciliary tips by interacting with IFT52, IFT57, and others. In KO rats, 1) the processes for ODA assembly and loading are defected, leading to the degradation of ODA components such as DNAI1 and DNAI2; 2) ODA is absent in motile cilia; and 3) enhanced anterograde trafficking may occur partially due to compensatory mechanisms, leading to the accumulation of anterograde trafficking-associated proteins such as IFT52 and IFT20 in motile cilia. Similar to ODA, IDA is also defected when PIH1D3 is deleted in KO rats.

In the ciliated cells of affected organs, PIH1D3 immunoreactivity was detected primarily in the base of cilia, suggesting that PIH1D3 is possibly involved in the loading and trafficking of cargo to cilia tips. Dynein arm pre-assembly is believed to take place in the cytoplasm, while the loading of assembled dynein arms occurs at the base of motile cilia. Consistent with previous findings (Dong et al., 2014; Olcese et al., 2017; Paff et al., 2017), our results show that PIH1D3 deficiency caused defects in both the outer and inner dynein arms. In PIH1D3-KO rats, depletion of the critical components from ODA and IDA was revealed by immunofluorescence staining, and defects in ODA and IDA structures were detected by electron microscopy. Biochemical analysis revealed that PIH1D3 interacted with the proteins involved in the assembly and trafficking of ODA and IDA. As illustrated in Figure 8, PIH1D3 interacts with the DNAAF family to facilitate assembling the cargos for ODA and IDA and interacts with IFT52 and IFT57 to initiate the trafficking of cargos to IDA and ODA. When PIH1D3 was depleted, the assembly of cargos and the initiation of cargo trafficking were negatively affected, leading to the depletion of ODA and IDA components in the tips of cilia. As a result, the cargo trafficking process was augmented for compensatory mechanisms, resulting in the increased distribution of transport proteins to cilia tips. The pathological manifestations observed in Pih1d3-KO rats exhibited a consistent association with motile cilia characterized by the classic 9 + 2 microtubule structure. These phenotypes manifested as mucociliary defects in respiratory tracts, compromised motile cilia functionality in spermatocytes leading to infertility, and impaired motile cilia in brain epithelial cells contributing to hydrocephalus. Notably, the expression of PIH1D3 was found to be localized to multi-ciliated cells, encompassing the ependymal epithelium lining of the ventricular surface of the brain, the choroid plexus residing within the ventricular lumen, the epithelial lining of the respiratory tract, and the efferent ducts in male reproductive organs. Consequently, our investigation was predominantly focused on elucidating the intricate molecular mechanism responsible for the dysfunction of motile cilia in the context of PIH1D3-related ciliopathy. Previous studies focused on the effects of PIH1D3 deficiency on ODA and IDA assembly (Dong et al., 2014; Olcese et al., 2017; Paff et al., 2017), but how PIH1D3 regulates dynein arm structure and function is not clear. Ciliogenesis consists of multiple steps including pre-assembly of dynein cargos in the cytoplasm, uploading of cargos onto IFT-train in the base body of cilia, ferrying cargos to the transition zone, and delivery of cargos to cilia tips through anterograde transport (Cho et al., 2018). Our data suggest that PIH1D3 is implicated in both the assembly and trafficking of ODA and IDA, providing a novel insight into PIH1D3 functions.

Preservation of function in mutated PIH1D3 affects the severity of disease phenotypes in patients (Olcese et al., 2017; Paff et al., 2017; Paff et al., 2018; Aprea et al., 2021; Lennon et al., 2022). Our PIH1D3-KO rats were generated by introducing a frameshift in exon 5 and inducing subsequent frameshift within exon 6, leading to the nonsense-mediated decay of mutant PIH1D3 transcripts. A full depletion of PIH1D3 in the KO rats induced robust phenotypes, including communicating hydrocephalus. The hydrocephalus phenotype is correlated with the deletion of PIH1D3 from the epithelial cells of ependyma and choroid plexus in the KO rats, although hydrocephalus was not reported in patients carrying a pathogenic mutation in PIH1D3 (Olcese et al., 2017; Paff et al., 2017; Paff et al., 2018; Aprea et al., 2021; Lennon et al., 2022). The difference in phenotypic expression between humans and rats possibly results from the difference in compensatory mechanisms in different species. The loss of PIH1D3 in ependyma and choroid plexus might be functionally compensated by the unknown genes of similar functions in humans.

Hydrocephalus is a prominent phenotype in PIH1D3-KO rats. X-linked hydrocephalus has an incidence of 1/30,000 male births (Wang et al., 2021). Our studies reveal a novel function for PIH1D3 in the circulation of cerebrospinal fluids. Immunofluorescent staining shows that PIH1D3 is robustly expressed in the ciliated cells of ependyma and choroid plexus in the rat brain. Increasing studies indicate that dysfunction in the ciliated cells of the ependyma and choroid plexus causes hydrocephalus (Banizs et al., 2005; Funk et al., 2015; Abdelhamed et al., 2018; Han et al., 2022). Hydrocephalus is categorized into communicating and non-communicating types, depending on whether there is a blockage in the brain ventricular system. Communicating hydrocephalus lacks such blockage and is typically caused by a deficiency in the driving force for fluid flow. CSF directional flow relies on external forces, with motile cilia playing a crucial role in propelling CSF within brain and spinal cavities. As dynein arms are essential for the bending movement of cilia (Satir and Christensen, 2007; Xu et al., 2023), PIH1D3 deficiency causes hydrocephalus by impairing the pre-assembly and trafficking of dynein arms in ciliated cells. Deletion of the *CCDC39* gene in KO mice causes neonatal hydrocephalus with abnormal motile cilia (Abdelhamed et al., 2018). The ciliated cells of ependyma mobilize the circulation of cerebrospinal fluids, whereas the ciliated cells of the choroid plexus produce and absorb cerebrospinal fluids in the brain (Lun et al., 2015; Faubel et al., 2016). The most ciliated cells of the choroid plexus exhibit the characteristics of motile cilia with the 9 + 2 microtubule configuration at the embryonic stage and retain the motile cilia characteristics at birth but gradually lose the motile cilia features in 2 weeks after birth (Narita et al., 2012; Damkier et al., 2013; Nonami et al., 2013). In rats, PIH1D3 is substantially expressed in the ciliated cells of the choroid plexus, and its deficiency causes defects in the cilia and subsequently impairs the function of the choroid plexus in equilibrating cerebrospinal fluid during embryonic and postnatal stages. The severe hydrocephalus in PIH1D3-KO rats is likely a combined effect of impairment to the ciliated cells of ependyma and choroid plexus. It is of high diagnostic and therapeutic value to identify the genes that compensate for the loss of *PIH1D3* in humans.

## Conclusion

This study generated and characterized PIH1D3-KO rats that reproduced the cardinal features of ciliopathy. A novel function of PIH1D3 in regulating cerebrospinal fluid was uncovered, and its pathological significance was defined. The study biochemically defined the mechanism by which PIH1D3 regulates cilial integrity and function through interacting with the proteins required for the pre-assembly and uploading of dynein arms. The resulting rat model is useful for studying the mechanisms and therapeutics of PIH1D3-associated ciliopathy, and the biochemical findings could improve our understanding of the pathomechanisms underlying diseases caused by PIH1D3.

## Data Availability

The original contributions presented in the study are included in the article/Supplementary Material; further inquiries can be directed to the corresponding authors.

## Abbreviations

PIH1D3, PIH1 domain-containing protein 3; TALEN, transcription activator-like effector nuclease; KO, knockout; WT, wild-type; ODA, outer dynein arms; IDA, inner dynein arms; SD, Sprague–Dawley; CSF, cerebrospinal fluid.
