# Supplementary material for: PIH1D3-knockout rats exhibit full ciliopathy features and dysfunctional pre-assembly and loading of dynein arms in motile cilia
Source: Front Cell Dev Biol. 2023 Oct 12;11:1282787. doi: 10.3389/fcell.2023.1282787 (PMC10601634; doi:10.3389/fcell.2023.1282787)
Supplement: Supplementary file 1 [file DataSheet1.PDF]

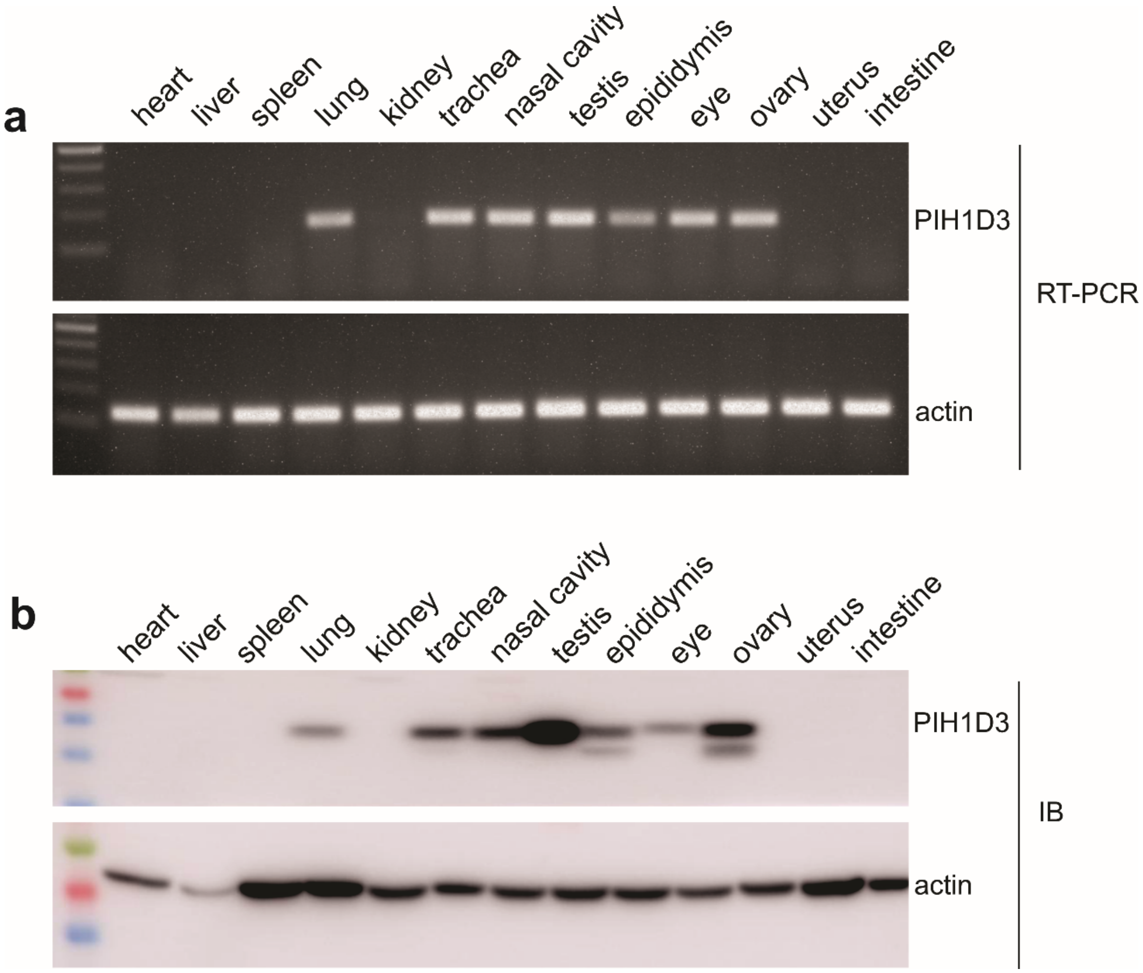

Supplemental Figure S1: Profiling the expression of PIH1D3 in postnatal rats. a) RT-PCR detected the expression of PIH1D3 mRNA in some of the tissues examined. **b)** Immunoblotting revealed the expression profile of PIH1D3 protein in some of the tissues examined. Equal loading of total proteins (20 µg/lane) was assessed by immunoblotting on the same membrane for actin. PIH1D3 was consistently detected in the same tissues examined at both the mRNA and protein levels. Tissues were dissected from wildtype male rats aging 23 days.

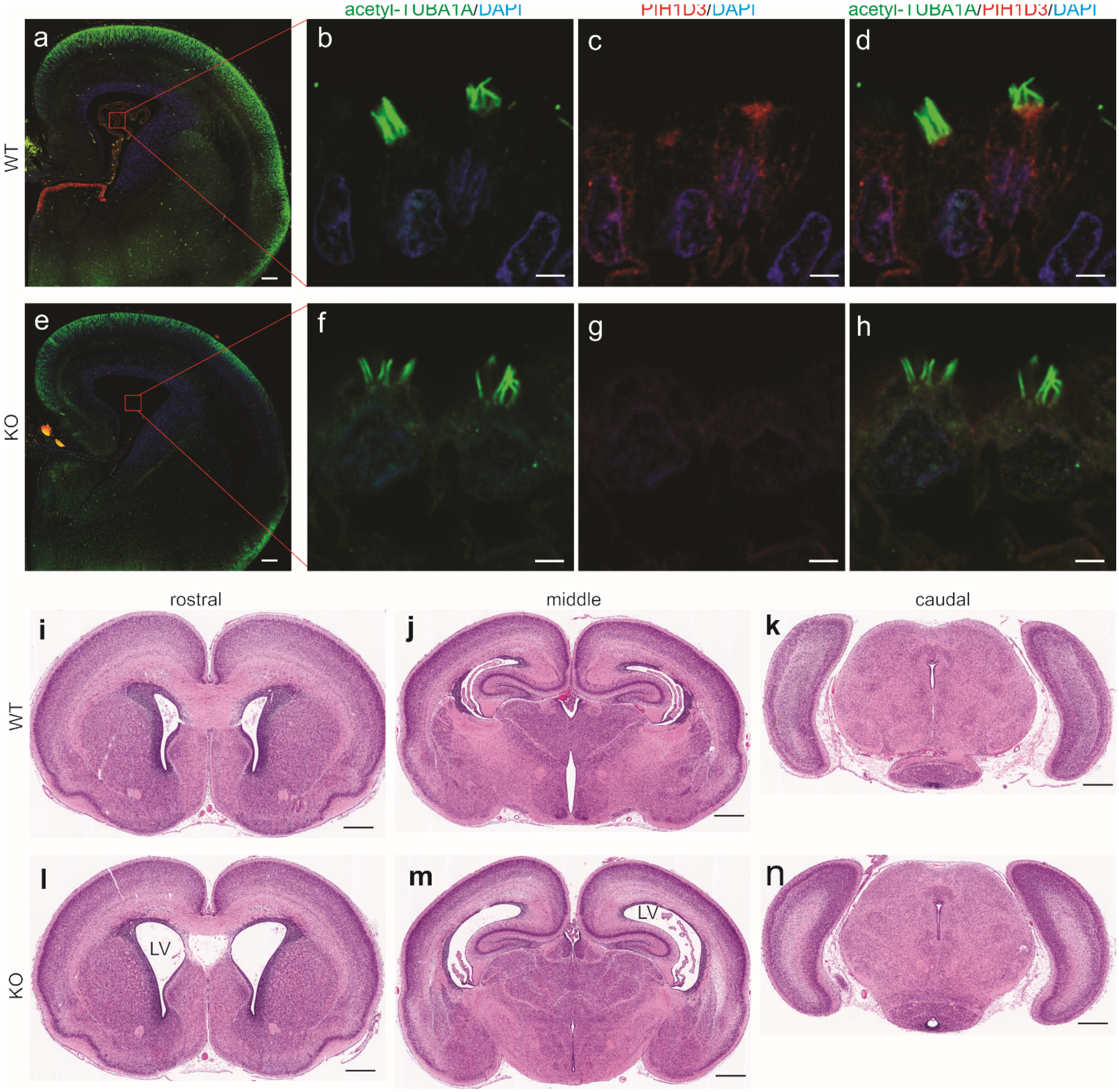

Supplemental Figure S2: Deletion of PIH1D3 in knockout rats causes hydrocephalus during embryonic development. a-h) Immunofluorescent staining for acetylated alpha-tubulin (acetyl-TUBA1A) and PIH1D3 reveals depletion of PIH1D3 proteins from choroid plexus epithelial cells in Pih1d3 knockout (KO) rats. KO rats and wildtype (WT) male littermates were examined at age of embryonic day 19. Scale bars: 100 µm (a, e) and 30 µm (b-d & f-h). **i-n)** HE staining of coronal brain sections reveals enlarged lateral ventricles (LV) in KO rat embryos at embryonic day 21. Scale bars: 150 µm.

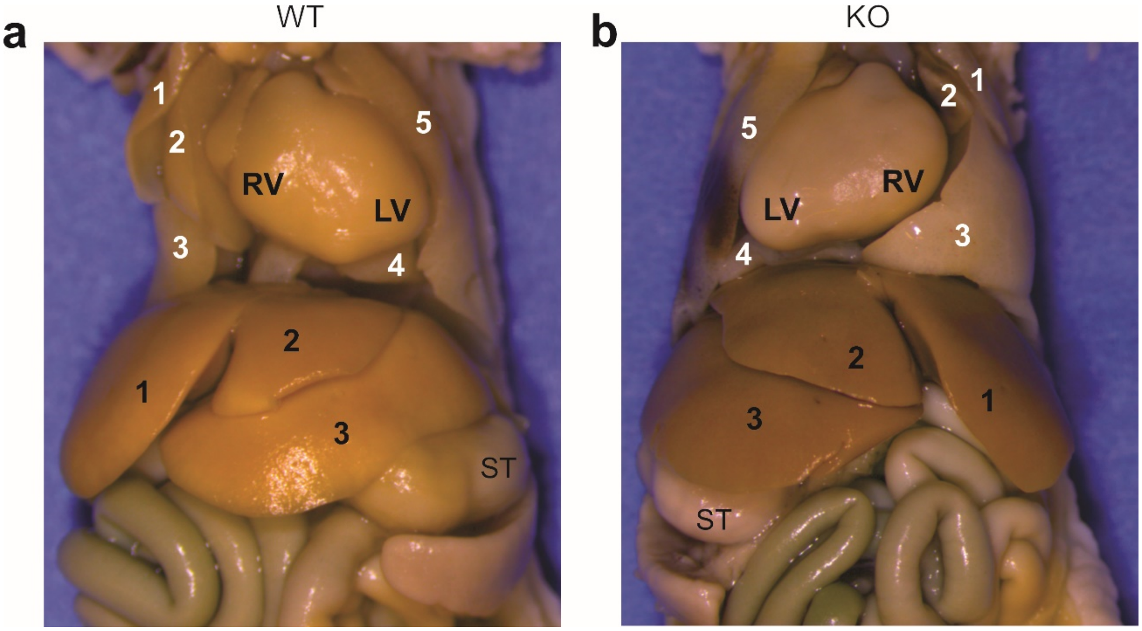

Supplemental Figure S3: Situs inversus revealed in PIH1D3 knockout (KO) rats. a, b) Photos taken of the chest and abdomen organs dissected from PIH1D3 KO rat and its wildtype (WT) littermate at the age of postnatal day 2. Organs in the chest and abdomen of PIH1D3 KO rat were positioned in a mirror image of WT rat anatomy. Numerical digits 1-5 in white color indicate the lobes of lung and numerical digits in dark color indicate the lobes of liver. Abbreviation: LV, left ventricle; RV, right ventricle; and ST, stomach.

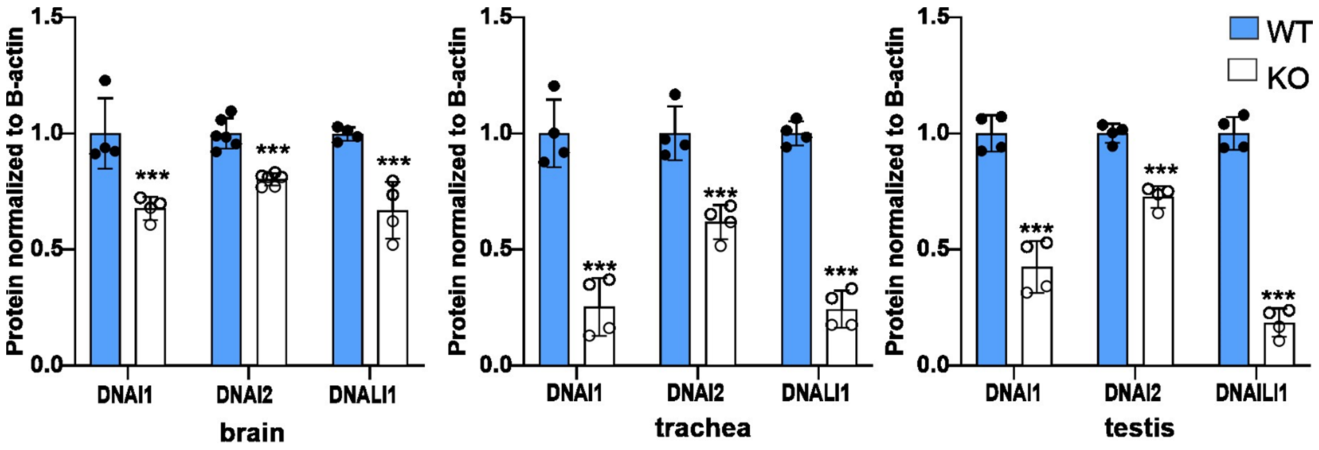

Supplemental Figure S4: The density of immunoblotting in Figure 6D was quantitated by measuring the intensity of each band using densitometry on a Koda imaging station. These values were calculated as ratios to the internal control actin. Four separate gels were analyzed for individual tissues from different rats. The results are presented as means ± SD (n = 4).

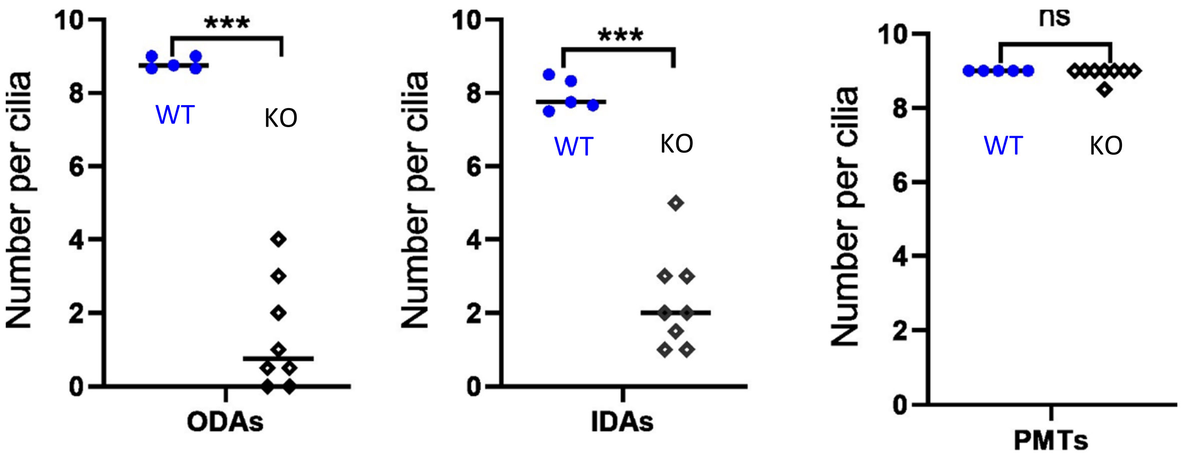

Supplemental Figure S5: Quantification of ODA, IDA, and peripheral doublet microtubules in the trachea of PIHI1D3 KO and WT rats. Outer dynein arm (ODA), inner dynein arm (IDA), and peripheral doublet microtubules (PMT) were examined on transmission electron microscopy (TEM) images. Representative TEM images were shown in Figure 6E-F. In total, 14 cilia and 10 cilia were counted for WT and KO rats, respectively. ** $p < 0.05$.

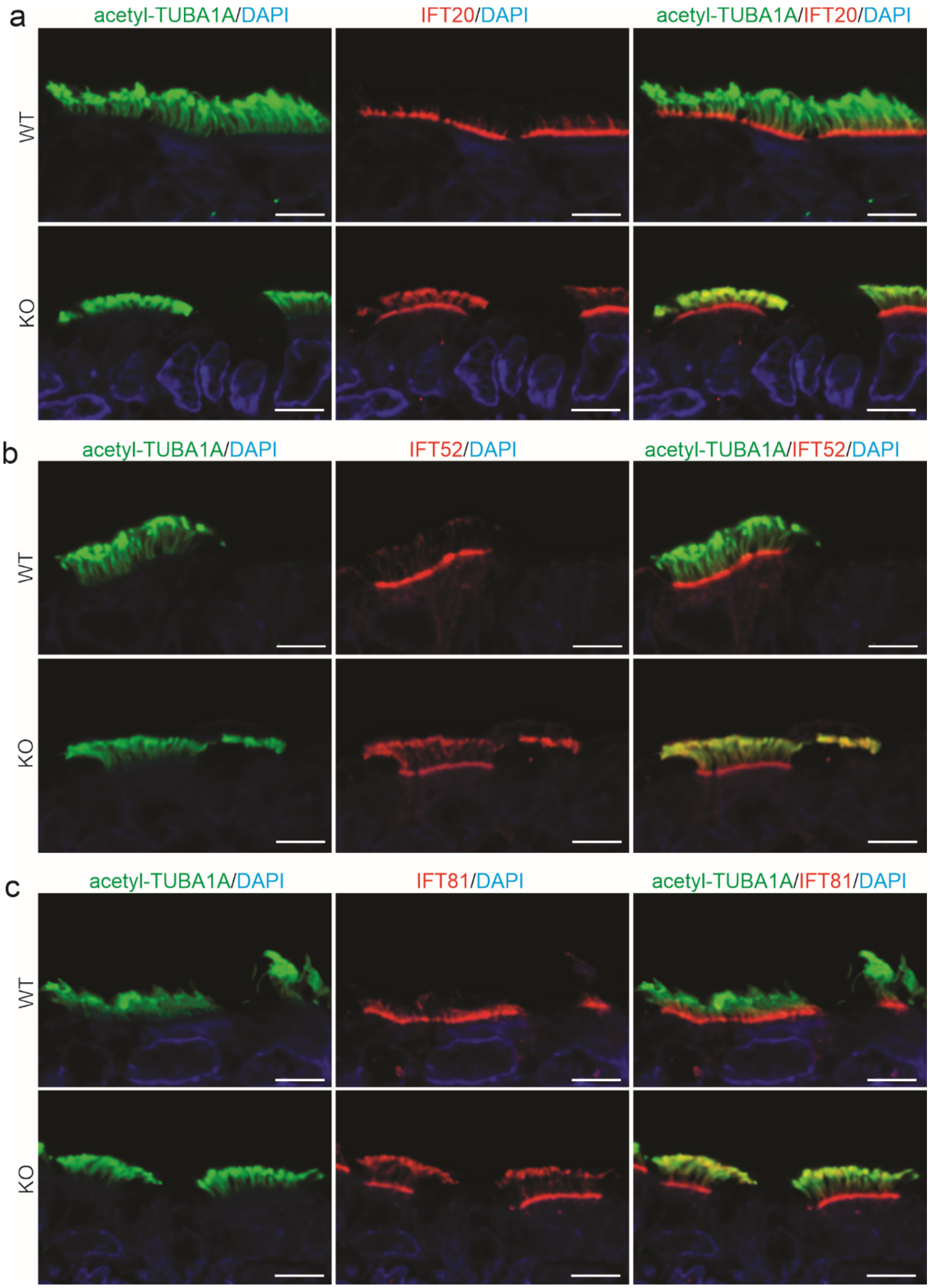

Supplemental Figure S6: Deletion of PIH1D3 in KO rat results in accumulation of intraflagellar transport proteins in the motile cilia of trachea. a-c) Confocal microscopy reveals the enhanced intensity of immunofluorescence staining for intraflagellar transport proteins including IFT20, IFT52, and IFT81 in the tips of motile cilia in KO rats as compared to wildtype (WT) littermates. Cell nuclei were labeled with DAPI and motile cilia were labeled with an antibody to acetylated alpha-tubulin (acetyl-TUBA1A). Sections of trachea were assessed by double-labeling immunofluorescence staining and the tissues were dissected from KO and WT littermates at the age of postnatal day 5. Scale bars: 5 μm.

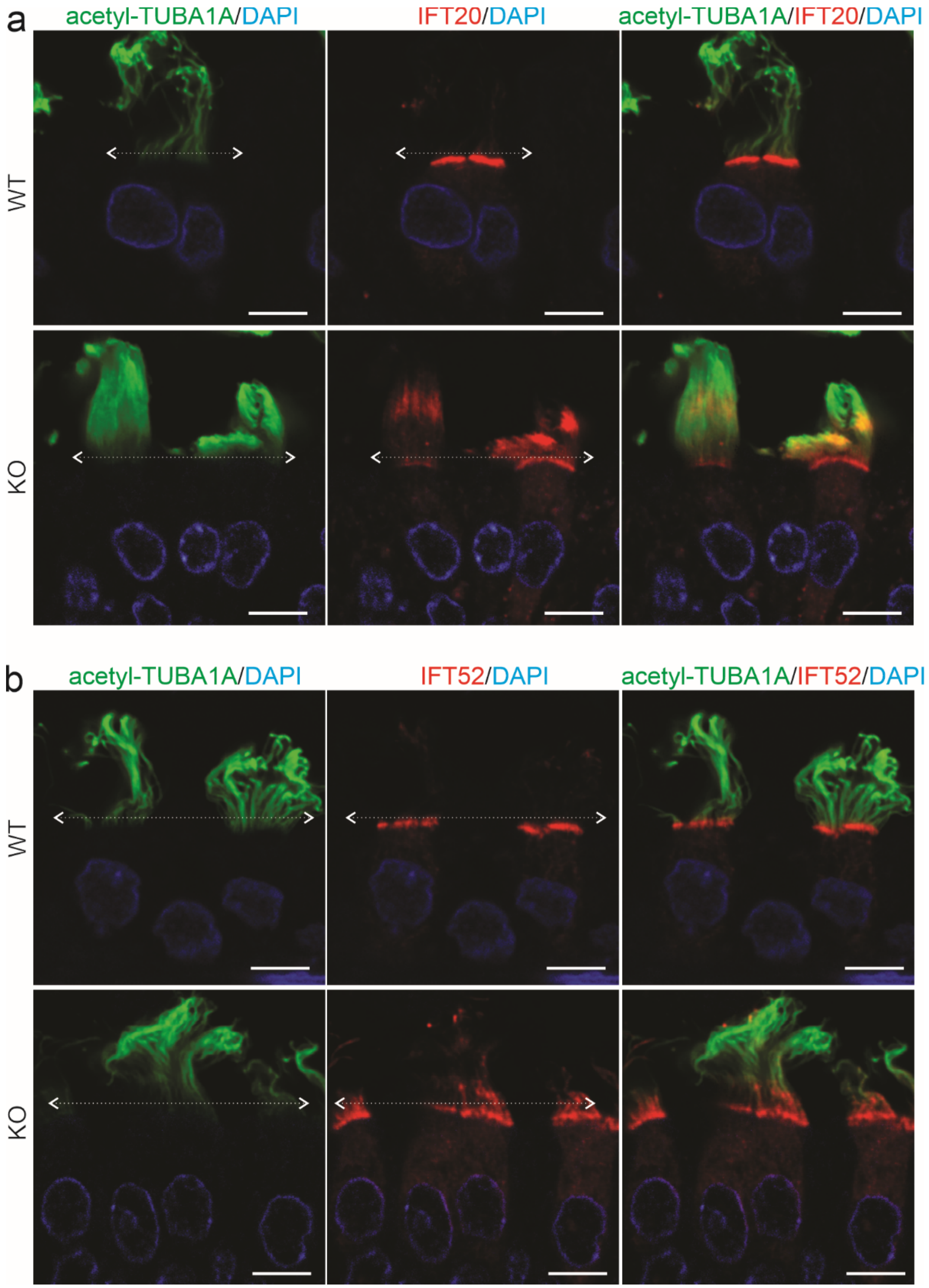

Supplemental Figure S7: Altered distribution of IFT20 and IFT52 in the motile cilia of efferent ducts in Pih1d3 KO rats. a-b) Confocal microscopy reveals the enhanced intensity of immunofluorescence staining for the intraflagellar transport proteins IFT20 and IFT52 in the tips of motile cilia in KO rats as compared to WT littermates. Cell nuclei were labeled with DAPI and motile cilia were labeled with an antibody to acetylated alpha-tubulin (acetyl-TUBA1A). Efferent ducts were dissected from KO and WT male littermates. Double arrowed lines marked the base of motile cilia. Scale bars: 5 µm.

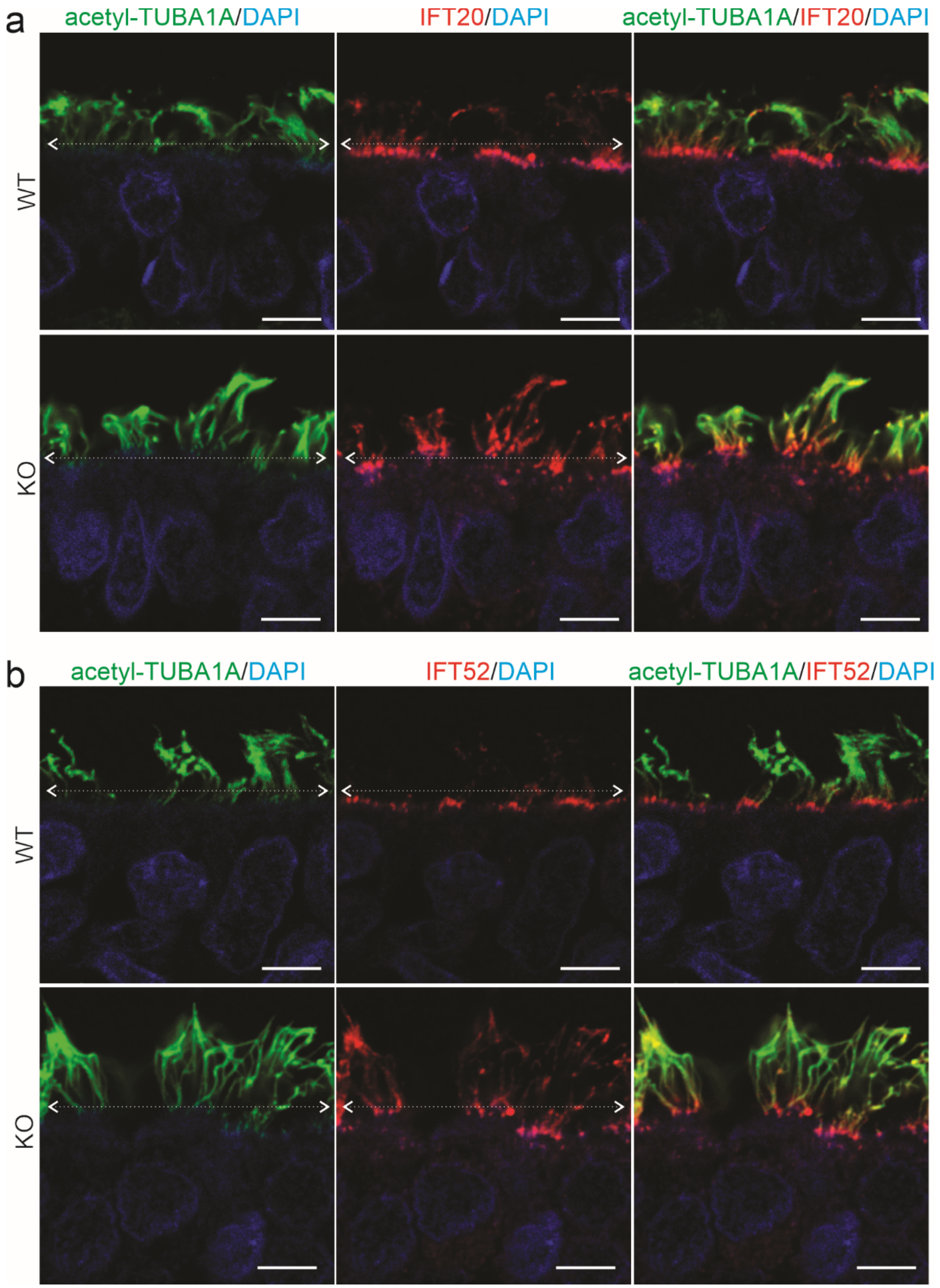

Supplemental Figure S8: Altered distribution of IFT20 and IFT52 in the motile cilia of brain ependymal epithelial cells in Pih1d3 KO rats. a-b) Confocal microscopy reveals the enhanced intensity of immunofluorescence staining for the intraflagellar transport proteins IFT20 and IFT52 in the tips of motile cilia in KO rats as compared to WT littermates. Cell nuclei were labeled with DAPI and motile cilia were labeled with an antibody to acetylated alpha-tubulin (acetyl-TUBA1A). Sections of forebrain through the ependymal cells of lateral ventricles were stained with antibody to IFT20 or IFT52. Tissues were dissected from KO and WT male littermates at postnatal day 5. Double arrowed lines marked the base of motile cilia. Scale bars: 5 µm.

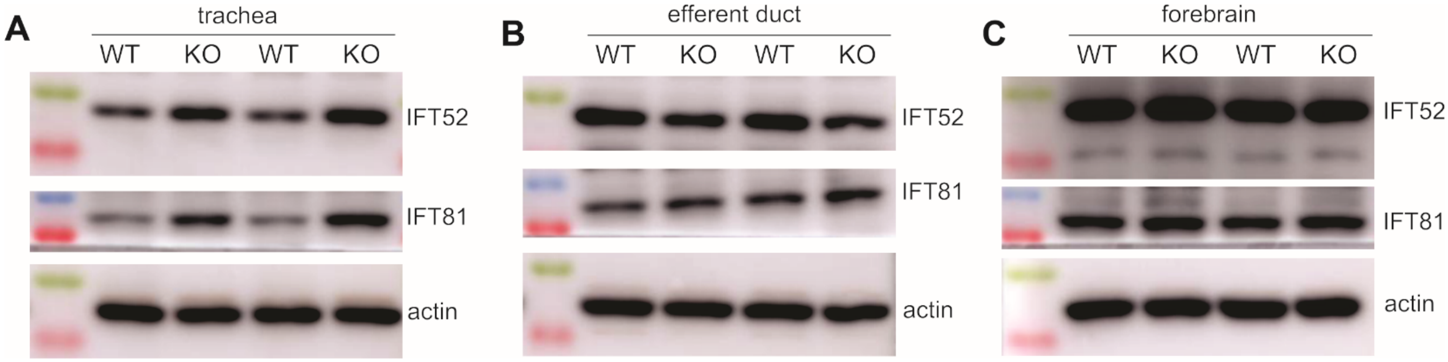

Supplemental Figure S9: Expression of intraflagellar transport proteins altered in the selected tissues of PIH1D3 KO rats. a) Immunoblotting shows that the expression of the intraflagellar transport proteins IFT52 and IFT81 increased in the trachea of Pih1d3 KO rats at the age of postnatal day 15 (P15). Each lane was loaded with 10 µg of total proteins. **b)** Immunoblotting revealed no significant change to the expression of IFT52 and IFT81 in the efferent ducts of Pih1d3 KO rats as compared to WT littermates. The staining density for IFT52 was slightly reduced in KO rats; however, the corresponding control actin also showed a lower density in the KO tissues. Tissues were dissected from postnatal rats at age P32 and each lane was loaded with 10 µg of total proteins. **c)** Immunoblotting shows a slight increase in the expression of IFT52 and IFT81 in the forebrain of Pik1d3 KO rats at age P5. Each lane was loaded with 10 µg of total proteins. Younger animals were used for dissecting forebrain because hydrocephalus destroyed brain tissues at later ages.
